# Defining the Violence Victim Phenomenon: A Qualitative Study Among Anesthesiology and Intensive Care Specialists

**DOI:** 10.3390/jcm15041503

**Published:** 2026-02-14

**Authors:** Pinar Ayvat, Ali Galip Ayvat

**Affiliations:** 1 Anesthesiology and Reanimation Department, Faculty of Medicine, Izmir Democracy University, 35140 İzmir, Türkiye; drpinarunde@yahoo.com; 2 Project Management Department, Izmir Project Agency, 35210 İzmir, Türkiye

**Keywords:** violence victim phenomenon, workplace violence, anesthesiology, second victim phenomenon, qualitative research, intensive care

## Abstract

**Background/Objectives**: Healthcare workplace violence has evolved into a global crisis, significantly impacting high-risk specialties. While the “Second Victim Phenomenon” (SVP) is well-established for trauma following medical errors, the specific psychological trauma resulting from intentional external aggression remains conceptually under-defined. This study aims to introduce and define the “Violence Victim Phenomenon” (VVP) by exploring the lived experiences of anesthesiology and intensive care specialists, providing a theoretical framework for this distinct clinical state. **Methods**: A qualitative study was conducted with ten anesthesiology and intensive care specialists using a semi-structured focus group discussion. The session was subjected to thematic analysis using MAXQDA software. The analysis focused on the nature of violence encountered, psychological and professional impacts, and the role of institutional support systems. **Results**: The thematic analysis identified six core dimensions of VVP: forms and trajectories of violence, vulnerability amplifiers, psychological and occupational sequelae, coping and containment strategies, expectations of institutional support, and pandemic-specific intensifiers. Participants described a trauma profile comparable to SVP in severity but distinct in its etiology, rooted in intentional harm and “institutional abandonment.” VVP is characterized by a profound sense of vulnerability, loss of professional dignity, and a perceived lack of legal and administrative protection. **Conclusions**: VVP represents a critical gap in current academic literature. Defining VVP allows for a more nuanced understanding of the trauma healthcare workers face due to intentional aggression. To mitigate VVP, healthcare institutions must move beyond basic security measures toward a “just culture” that provides structured psychological, legal, and managerial support, recognizing clinicians as victims of systemic failure.

## 1. Introduction

Healthcare workplace violence has emerged as a critical global public health and occupational safety crisis, threatening the core of healthcare delivery systems worldwide. According to the World Health Organization and recent large-scale meta-analyses, healthcare professionals are approximately five times more likely to experience violence in the workplace than employees in any other service sector, with global prevalence rates exceeding 60% across various clinical settings [1,2]. This phenomenon is not limited to physical assaults; it manifests through a complex spectrum including verbal abuse, psychological intimidation, and systemic hostility, all of which have intensified in the post-pandemic era [3]. Beyond being an isolated act of aggression, workplace violence serves as a profound source of professional trauma that destabilizes the psychological well-being of clinicians, compromises patient safety, and triggers significant organizational challenges such as burnout and high turnover rates [4,5].

The psychological impact of traumatic events on healthcare workers is not a new phenomenon. When both the patient and healthcare worker are harmed due to an error, complication, or unexpected clinical outcome during patient care, the emotional and professional impact experienced by the healthcare workers has been defined in the literature as the Second Victim Phenomenon (SVP) [6]. SVP highlights that healthcare professionals may experience feelings of guilt, shame, fear, and a loss of professional self-esteem after such an event, thus emphasizing the need for psychological and organizational support [7]. Therefore, SVP has made the provision of support for healthcare workers an essential aspect of modern healthcare systems. While the SVP framework has revolutionized our understanding of clinician trauma, it remains fundamentally anchored in the context of unintentional clinical error or patient harm. In SVP, the clinician’s primary psychological burden is guilt—the ‘what have I done?’ question. However, when a specialist is targeted by intentional aggression, the psychological pivot shifts from guilt to a sense of existential vulnerability and ‘institutional betrayal.’ Current literature lacks a unifying term that captures this transition from a clinician who ‘made a mistake’ to a clinician who has been ‘intentionally violated’ by the very community they serve. This conceptual gap leaves healthcare organizations without targeted protocols for the latter.

A similar interaction occurs when healthcare workers are subjected to violence. Systematic reviews and meta-analyses have shown that employees who experience violence exhibit significantly higher levels of anxiety, depression, insomnia, burnout, and a tendency to leave their jobs [8,9]. In this context, violence is not only an act of aggression but also a traumatic life event that threatens the healthcare worker’s professional identity and psychological integrity. Therefore, the need for professional and psychological support after experiencing violence is a natural and inevitable process, as seen in the SVP. The Violence Victim Phenomenon (VVP) that we propose in this study retains the core human aspect of SVP, while expanding the source of the phenomenon and considering all forms of violence—verbal, physical, structural, and symbolic—as triggers for the healthcare worker’s trauma. VVP assumes that healthcare workers can be traumatized not only by clinical care processes but also by the institution’s operations, security culture, and societal attitudes toward healthcare workers.

The severity of this issue is particularly evident in high-pressure clinical environments. Studies conducted in Turkey present an alarming picture, indicating that healthcare personnel working in emergency departments, intensive care units, and outpatient clinics encounter violence at exceptionally high rates [10]. Given that anesthesiology and reanimation specialists often operate in these high-risk areas, they are frequently the primary targets of both physical and verbal aggression. Despite the prevalence of these incidents, the qualitative dimensions of how such violence reshapes the professional identity and psychological health of these specialists remain under-explored. The COVID-19 pandemic served as a catalyst for this crisis, fundamentally altering the social contract between the public and healthcare workers. Recent studies suggest a ‘post-hero narrative shift,’ where the initial societal appreciation for frontline workers has morphed into frustration and scapegoating in the face of resource scarcity and systemic delays [11]. For anesthesiology specialists—who frequently occupy the physical and emotional interface of life-and-death decisions—this shift has resulted in a heightened risk of complex trauma [12]. Defining VVP is therefore not merely a semantic exercise but a necessary step in addressing the specific occupational health challenges of the post-pandemic clinical landscape.

This study aims to explore the conceptualization of VVP within a qualitative analysis framework, based on data obtained from focus group discussions with physicians working in intensive care and anesthesia departments. The study’s goal is to reveal the psychological, professional, and ethical impacts of violence in healthcare; to develop a unique theoretical framework that can explain these impacts; and to emphasize the necessity of institutional support mechanisms.

## 2. Materials and Methods

Focus group discussions are conversations conducted with a group on a specific topic and are of significant importance in qualitative research. They can be defined as a series of planned discussions aimed at understanding the thoughts, experiences, interests, tendencies, perceptions, emotions, attitudes, habits, and views of participants regarding a pre-determined topic. The goal of focus group discussions is to gather in-depth, detailed, and multidimensional qualitative information about participants’ perspectives, experiences, interests, and emotions on the topic at hand. After obtaining ethics committee approval, the focus group discussion was conducted for this research.

Participant Selection and Data Collection:

Participants were selected using purposive sampling to ensure a cohort with significant experience in high-risk clinical settings. Inclusion criteria required participants to be board-certified specialists in anesthesiology and intensive care with at least 5 years of clinical practice and active involvement in bedside patient care. Exclusion criteria included physicians in training (residents) or those primarily in administrative roles without regular patient contact. Recruitment was conducted through professional networking groups at three hospitals. Potential volunteers were provided with a brief overview of the study’s aim. A total of ten individuals who met the criteria and volunteered to participate were included. All participants held the same academic level and served as specialists in their respective institutions to ensure a homogenous professional perspective

A single focus group session was conducted in a physical conference room and lasted 80 min. The discussion was led by the lead researcher (MD, Associate Professor of Anesthesiology and Reanimation), and no external observers were present during the session to maintain a private environment conducive to open disclosure. The session was audio-recorded for subsequent transcription and analysis. Participants were anonymized by numbering them (Doctor 1: 51 years old, male; Doctor 2: 50 years old, male; Doctor 3: 49 years old, female; Doctor 4: 38 years old, female; Doctor 5: 40 years old, female; Doctor 6: 45 years old, male; Doctor 7: 57 years old, male; Doctor 8: 53 years old, male; Doctor 9: 59 years old, female; Doctor 10: 60 years old, female). The data collection was based on the main topics in the semi-structured interview form. Ten questions were asked during the focus group interview (Table 1), which were conversation-style, open-ended questions suitable for everyday language [13]. Special attention was given to ensuring the questions were clear and understandable. The questions were developed through a systematic preliminary process to ensure the questions were both clinically relevant and theoretically grounded. Initially, the lead researcher drafted a pool of questions based on a literature review of SVP and existing frameworks for healthcare workplace violence. To refine these questions, an expert panel was convened, consisting of two senior anesthesiology and intensive care specialists with over 20 years of experience and one academic researcher specializing in qualitative methodologies. This panel evaluated the questions for clarity, depth, and the potential to elicit data regarding VVP. The final ten questions were selected after achieving consensus among the panel members.

Data Analysis:

The data recorded during the interview were transcribed verbatim by the lead researcher. Data were analyzed using thematic content analysis, a systematic method used to identify, analyze, and report patterns (themes) within data. This approach was chosen for its ability to provide a descriptive and detailed account of the specialists’ experiences regarding workplace violence. The analysis followed a structured six-step process [14]:

Familiarization with the Data: The transcripts were read multiple times to achieve immersion and identify initial patterns.

Generating Initial Codes: Meaningful segments of text were identified and assigned codes representing the core concepts.

Searching for Themes: Codes were sorted and grouped into broader themes that reflected recurring patterns across the focus group discussion.

Reviewing and Refining Themes: The candidate themes were reviewed against the original transcripts to ensure they accurately represented the data.

Defining and Naming Themes: Clear definitions and names were developed for each theme to ensure conceptual clarity.

Writing the Report: Findings were presented through a coherent narrative.

The coding process was performed by two independent coders (the authors) to enhance the reliability of the findings. Following independent coding, the researchers met to compare their results; any disagreements in code application or thematic grouping were resolved through in-depth discussion until a 100% consensus was achieved. While the final themes were derived from the consensus of the research team, participant validation of the transcripts was not conducted to preserve the raw, immediate nature of the focus group’s collective narrative. The most frequently repeated and emphasized phenomena within the data were coded and grouped into themes [13]. The main aim here was to focus on the relationships that explain the obtained data [15]. MAXQDA 20 software was used in the data analysis process. MAXQDA facilitates a systematic interpretation of the obtained data. The adequacy of the sample size was justified based on the principle of thematic saturation. Given the homogeneous nature of the participant group—all of whom were specialists sharing similar professional backgrounds and clinical environments—thematic saturation was achieved during the analysis of the 80 min session. Saturation was confirmed when no new conceptual categories or dimensions of VVP emerged, and the identified themes became repetitive across the dataset. The reporting of this study follows the Consolidated Criteria for Reporting Qualitative Research (COREQ) guidelines (see Supplementary Materials, File S1).

## 3. Results

A thematic content analysis of the focus group transcripts yielded six overarching themes and twenty subthemes that collectively illustrate the multifaceted nature of violence within healthcare environments. The emergent construct, VVP, encapsulates clinicians’ experiences of direct, structural, and vicarious violence and delineates its personal, organizational, and systemic implications (Table 2).

1. Forms and Trajectories of Violence in Care

Participants described violence as a persistent and multidimensional phenomenon. Verbal aggression—often expressed through shouting, blame, and humiliation—was reported as an “expected” aspect of daily work, particularly after critical incidents. Over time, its normalization eroded professional dignity and trust between healthcare providers and patients’ families.

Physical threats and near-miss assaults, such as throwing objects or physical intimidation, were perceived as “turning points” that triggered immediate fear and physiological distress.

Beyond interpersonal aggression, institutional and structural violence emerged as a dominant theme. Participants linked unsafe infrastructure, resource shortages, and governance failures to a sense of systemic betrayal: “When nothing changes despite repeated warnings, that’s also violence.”

Finally, vicarious or ambient violence—witnessing aggression or patient suffering—elicited comparable emotional exhaustion even among those not directly targeted.

2. Vulnerability Amplifiers and Exposure Pathways

Clinicians identified multiple mechanisms that heighten exposure to violence. Frontline proximity during invasive procedures positioned anesthesiologists as frequent targets of frustration and grief. Information asymmetry arising from shift changes or inconsistent communication fueled mistrust among patients’ relatives.

Governance gaps—the absence of formal protocols or inconsistent managerial responses—intensified feelings of helplessness. Participants also highlighted environmental design deficits, such as unsecured corridors and unrestricted visitor access, which created unsafe working conditions.

3. Psychological and Occupational Sequelae

Exposure to violence produced both acute and chronic repercussions. Acute stress reactions—tremors, insomnia, and intrusive thoughts—were immediate responses, while moral injury and professional cynicism developed as staff perceived abandonment by their institutions. Several participants expressed defensive practice, such as ordering unnecessary tests or over-documenting, to prevent confrontation or litigation.

Violence also permeated personal lives: emotional numbing and irritability contributed to spillover into home environments, weakening social and family relationships.

4. Coping, Containment, and Informal Safety Nets

Most participants lacked formal support mechanisms and relied on peer debriefing as their primary coping strategy. Sharing experiences with trusted colleagues provided emotional validation and practical reassurance. Boundary-setting and redirection—for instance, advising aggressive relatives to use formal complaint channels—served as protective tactics.

Personal rationalization and normalization of violence (“the system is broken, anyone would fail here”) were frequent self-defense mechanisms, although these risked reinforcing professional disengagement and chronic burnout.

5. Expectations of Support and System Obligations

Participants articulated a strong demand for institutional accountability through a three-tier rapid response model: (1) immediate psychological counseling, (2) real-time legal assistance, and (3) visible managerial protection, including the right to withdraw from threatening encounters.

The need for security and access control—such as visitor restrictions, entry screening, and trained de-escalation teams—was consistently emphasized. Moreover, a just culture approach to violence reporting was considered essential for systemic learning and prevention: “If incidents were discussed like medical errors, we could learn instead of hiding.”

6. Pandemic-Specific Intensifiers

Violence intensified during the COVID-19 pandemic. Resource scarcity, shifting guidelines, and equipment rationing (e.g., bronchoscopy restrictions) placed clinicians in untenable positions that often provoked conflict. Public stigma and misinformation generated additional hostility—some participants reported being treated as “carriers” or “outsiders” in their communities. These contextual stressors amplified the scope and visibility of VVP.

**Figure 1:**
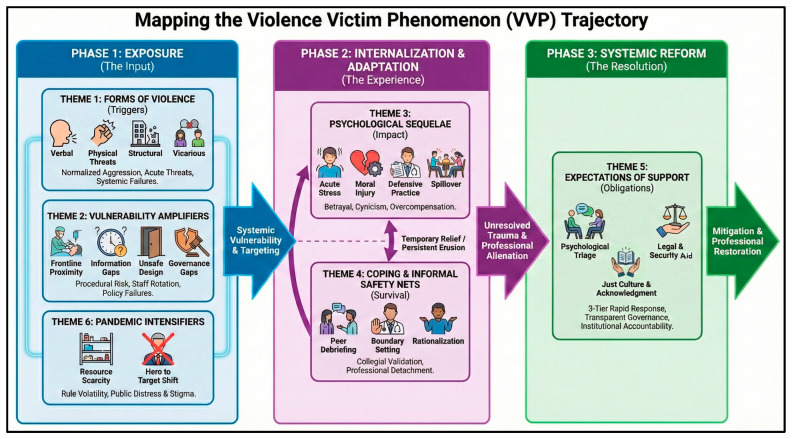
The conceptual framework of VVP.

The relationship between these themes and the overall trajectory of VVP is illustrated in the conceptual framework (Figure 1). This model demonstrates how external catalysts and clinical vulnerabilities converge with institutional factors to define the specialist’s experience.

## 4. Discussion

VVP proposed in this study offers a new conceptual framework that interprets the violence experienced by healthcare workers not as an isolated event, but as a multilayered process reproduced at systemic, institutional, and societal levels. International systematic reviews on violence in healthcare demonstrate that a large proportion of healthcare workers—ranging from 40% to 90% depending on the study—experience violence at some point in their professional lives [2,4]. This prevalence suggests that violence is not an exceptional incident but a chronic and structural component of healthcare delivery. At this point, VVP diverges from individual-centered explanations by presenting an integrated perspective that also encompasses the institutional and societal conditions underlying violence.

The findings of this study show that violence in healthcare settings does not occur randomly; rather, it emerges within an environment continuously shaped by specific structural vulnerabilities. Meta-analytic evidence indicates that understaffing, excessive workload, long waiting times, disorganized physical environments, and ineffective communication mechanisms are among the strongest predictors of violence [2,8]. Studies conducted in Turkey confirm this pattern, reporting that factors such as noncompliance with administrative procedures, insufficient visitor control, and dissatisfaction related to service quality often trigger violent incidents [10]. These findings align with Galtung’s [16] concept of structural violence. The harm experienced by healthcare workers often does not stem from a direct physical attack; rather, it originates from institutional deficiencies, ineffective governance structures, and security gaps. For this reason, VVP positions violence not solely as the perpetrator’s behavior but as a direct consequence of the institution’s failure to protect its employees.

When examining the psychological effects of violence, the findings of this study show complete concordance with the global literature. Violence is known not only to act as an acute stressor but also to cause deep and lasting psychological harm in healthcare workers. Among these effects, moral injury is considered one of the most devastating consequences. As defined by Litz et al. [17], moral injury develops when individuals feel betrayed by authority figures. Participants’ statements indicating a lack of institutional support after violent incidents demonstrate that this mechanism operates clearly in the context of healthcare workers. Indeed, strong evidence shows that healthcare workers exposed to violence experience increased levels of depression, anxiety, burnout, and intentions to leave their jobs [9,18]. For this reason, VVP conceptualizes violence not only in terms of the impact of physical or verbal assaults but also through the lens of the “secondary trauma” caused by institutional indifference and silence.

In this context, VVP is proposed as a novel conceptual framework that captures the multifaceted impact of violence on healthcare professionals, encompassing psychological, ethical, and organizational dimensions. Importantly, although VVP is introduced as a distinct phenomenon, it shares a conceptual foundation with the well-established SVP. In both cases, healthcare workers experience secondary trauma following disruptive events that challenge their professional identity, emotional stability, and sense of safety. While SVP traditionally focuses on the psychological consequences of unintended patient harm or adverse clinical events, VVP extends this perspective by shifting the source of trauma to external factors such as verbal abuse, physical assault, societal stigmatization, and institutional neglect. This parallel underscores that trauma in healthcare is not exclusively linked to medical errors but may also arise from hostile interactions and unsafe working environments. Recognizing this shared ground highlights the necessity for institutional support systems that address not only post-error recovery, as emphasized in SVP literature, but also post-violence recovery processes, which constitute the core of VVP framework.

The core defining attributes of VVP that distinguish it from existing frameworks are intentionality and systemic betrayal. Unlike SVP, where trauma arises from unintended clinical outcomes, VVP is rooted in the deliberate aggression of a human agent (the perpetrator). This intentionality transforms the psychological impact from ‘guilt’ (common in SVP) to a ‘sense of vulnerability’ and ‘moral injury.’ Furthermore, VVP is characterized by institutional abandonment, where the trauma is not just the act of violence itself, but the perceived failure of the organization to provide a safe environment or post-incident protection. These attributes delineate VVP as a specific occupational phenomenon rather than a generalized stress response.

One of the less visible dimensions of violence—underreporting—also forms a foundational component of the theoretical structure of VVP. In many countries, including Turkey, only a very small proportion of violent incidents are formally reported. In the study by Sari et al. [10], which analyzed cases through the White Code system, only 1.2% of incidents were officially documented. International data reveal a similar pattern, indicating that violence often remains concealed due to bureaucratic barriers, beliefs that reporting will be ineffective, lack of managerial support, and the internalization of violence as “a normal part of the job” [19,20]. This institutional silence not only amplifies the invisibility of violence but also deepens the trauma experienced by healthcare workers, reinforcing the persistence of VVP.

The COVID-19 pandemic increased both the visibility and the frequency of violence in the healthcare field globally, clearly demonstrating how dynamic VVP is. During the pandemic, psychological and physical violence against healthcare workers rose significantly; reports indicate that stigmatization, social exclusion, and anger toward hospital policies often escalated into aggression [8,21]. This shows that VVP is shaped not only by institutional structures but also by social perceptions and societal dynamics. In times of crisis, violence becomes not only a threat within the hospital but a symbolic threat directed at the identity of healthcare workers within society at large.

An increasingly accepted perspective in the literature is that violence cannot be resolved solely through individual responses or security measures. International guidelines published by the WHO and ILO also recommend a holistic, multi-level systems approach to combating violence in healthcare. This approach emphasizes that such efforts must include physical safety measures, effective communication systems, rapid psychological, legal, and administrative support mechanisms following violent incidents, and—most importantly—learning-oriented institutional processes grounded in the principle of a “just culture” [22]. VVP aligns precisely with this need, asserting that violence must be understood not merely as an event-based problem but as a systemic issue.

While VVP is a distinct conceptual state, it overlaps with several established psychological constructs. The symptomatic profile described by participants—including insomnia, tremors, and intrusive thoughts—parallels the diagnostic criteria for Post-Traumatic Stress Disorder [23]. However, VVP serves as the specific occupational ‘trigger’ for these symptoms within the healthcare context. Similarly, there is a significant overlap with burnout [2]; we posit that VVP acts as a potent intensifier of emotional exhaustion and depersonalization, often serving as the ‘breaking point’ that leads to professional turnover. Finally, VVP is inherently linked to moral injury, specifically the psychological distress resulting from the betrayal of ‘what’s right’ by those in positions of authority [24]. By recognizing these overlaps, VVP provides a more targeted framework for clinical and administrative interventions.

This study developed VVP as a new conceptual framework to explain healthcare workers’ experiences of violence. VVP defines violence not simply as a behavior but as a phenomenon shaped by the interaction of institutional, psychological, and societal processes. This framework indicates that healthcare institutions should not limit their efforts to increasing security measures alone; rather, they must develop comprehensive policies that encompass employee safety, organizational ethics, and psychological well-being. The key contribution of VVP to the literature is its reconceptualization of violence not merely as an immediate experience of the victim but as an ethical and safety issue that permeates the entire organizational structure. In this respect, VVP offers a guiding framework for future research, practical interventions, and policy development aimed at preventing violence in healthcare.

This study has several strengths and limitations that should be considered when interpreting the results. A primary strength is the high degree of professional homogeneity; all participants were anesthesiologists and intensive care specialists with significant clinical experience. This ensured a high level of “information power,” allowing for a deep, specialized exploration of VVP that would not have been possible in a more heterogeneous group. Furthermore, the use of two independent coders and adherence to the COREQ reporting guidelines enhance the reliability and transparency of the findings.

However, several limitations must be acknowledged. First, the study relied on a volunteer sample. This may have introduced a self-selection bias, where clinicians who have experienced more severe or frequent violence were more likely to participate, potentially skewing the findings toward more traumatic narratives. Second, while the focus group interview guide was validated by an expert panel, the nature of semi-structured focus groups can lead to response bias, where participants may feel influenced by the group dynamic or the facilitator’s prompts. Third, the sample size of ten participants, while sufficient for reaching thematic saturation in this specific professional group, limits the statistical generalizability of the findings. Finally, the study was conducted within tertiary care hospitals in a single geographical region, and cultural or legal differences in other healthcare systems may influence the manifestation and perception of VVP. Future research should involve larger, multi-regional samples to validate VVP framework across diverse clinical and cultural contexts.

## 5. Conclusions

The common ground between SVP and VVP is that, in both cases, the healthcare worker experiences secondary trauma and requires institutional support. In SVP, the trauma originates from a clinical event, whereas in VVP, it arises from external factors such as patient or relative behaviors, societal prejudices, or institutional neglect. In both situations, the healthcare worker is not merely a passive observer; they carry a significant emotional burden, their professional performance is affected, and without adequate support mechanisms they become more vulnerable to burnout and job turnover. Defining VVP extends the traditional frameworks that focus solely on medical errors and incorporates post-violence recovery processes into the core responsibilities of healthcare institutions. Therefore, this study introduces VVP as a distinct theoretical framework for understanding the occupational trauma experienced by anesthesiology and intensive care specialists. Our results demonstrate that VVP is not merely a reaction to isolated acts of aggression but a multilayered process rooted in intentionality and systemic betrayal.

The findings support three primary conclusions:

Violence is Systemic, Not Just Interpersonal: The identification of “structural violence” and “governance gaps” in our thematic analysis confirms that VVP is exacerbated by safe-working failures and institutional neglect, rather than just individual perpetrator behavior.

Institutional Abandonment is the Core Trauma: Consistent with the participants’ reports of feeling like “targets” without legal or administrative protection, the core of VVP is the perceived failure of the organization to uphold its duty of care. This distinguishes VVP from SVP, where trauma is rooted in internal guilt over medical errors.

A Shift to “Just Culture” is Essential: To mitigate VVP, healthcare institutions must move beyond basic security toward a “just culture” model. This includes structured, three-tier support—psychological, legal, and managerial—that treats clinicians as victims of systemic failure rather than individual targets.

By defining VVP, this study provides the conceptual language necessary for healthcare leaders to develop targeted intervention protocols, ultimately protecting the psychological integrity and professional longevity of specialists in high-risk clinical environments.

## Figures and Tables

**Table 1:** Open-ended questions asked during the focus group discussion.

Subject	Focus Group Discussion Questions
Experience and trajectory	Can you describe your most impactful experience with workplace violence in an anesthesia or ICU setting? How do you distinguish between “routine” verbal aggression and physical threats in your daily workflow? What role did the COVID-19 pandemic play in changing the nature or intensity of the violence you face?
Professional Impact	4. How do you perceive the “normalization” of violence within the medical community? 5. How has exposure to violence affected your professional identity and “defensive medicine” practices?
Psychological & Personal Sequelae	6. What are the immediate physiological and psychological responses you experience after a violent event? 7. Does the trauma of workplace violence spill over into your personal and family life? If so, how?
Institutional Context & Solutions	8. In what ways do you believe institutional factors (e.g., security, staffing levels) contribute to these incidents? 9. What are your specific expectations for psychological, legal, and administrative support from your institution? 10. What institutional changes do you believe are necessary to transition toward a “just culture” regarding violence?

**Table 2:** Codebook for thematic analysis.

Main Theme	Subtheme	Analytic Definition	Representative Quotation
1. Forms and Trajectories of Violence	Verbal aggression normalized over time	Recurrent verbal assaults—shouting, blaming, or insulting language—that become routine and gradually accepted as part of clinical work.	“We get used to being yelled at after every loss. Even if they don’t shout at me directly, I go home with that noise in my head.” “Insults are now just background noise in the ICU; we’ve learned to ignore them just to finish the shift.”
	Acute physical threats and near-miss assaults	Direct bodily attacks or physical intimidation by patients or relatives that endanger healthcare workers’ safety.	“He threw the monitor stand; it passed just next to me. I kept shaking the whole shift but still had to continue working.” “A relative grabbed my collar because I wouldn’t let them into the sterile zone. I felt completely trapped and defenseless.”
	Structural or institutional violence	Harm produced by unsafe working conditions, resource shortages, or managerial neglect that exposes staff to risk.	“We warn that there’s no security at the door, no negative pressure room, but nothing changes. That’s also a kind of violence.” “We warn the administration about the lack of safety measures, but nothing changes. The system leaves us exposed.”
	Vicarious/ambient violence	Emotional toll of constant exposure to death, deterioration, and aggression around others.	“Even when the fight isn’t mine, witnessing it makes me feel unsafe in my own workplace.” “Witnessing a colleague being attacked makes me feel unsafe. You realize the next hit could easily be for you.”
2. Vulnerability Amplifiers and Exposure Pathways	Frontline proximity & procedural risk	Frequent close contact during high-risk interventions (e.g., airway, invasive monitoring) increases exposure to aggression and infection.	“Anesthesiologists are always at the patient’s face—intubation, suction, bronchoscopy—so we face the family’s anger first.” “When you are the one holding the syringe or the tube, you are the easiest target for their grief.”
	Information asymmetry & rotating staff	Inconsistent communication with families due to shift changes, causing mistrust and confrontation.	“Every day another doctor gives another story; they think we’re hiding something.” “Relatives hear different things from different doctors. When the information is inconsistent, their confusion turns into rage.”
	Governance gaps	Absence of clear institutional protocols for managing violence or complaints.	“There’s no rule—some managers intervene, some don’t. It depends on luck.” “The administration tells us to be ‘more empathetic’ while we are actively being threatened. There is no clear protocol for protection.”
	Environmental design deficits	Physical layout enabling intrusion or unsafe contact (no controlled entries, inadequate security).	“Anyone can enter the ICU corridor; sometimes ten relatives at once.” “The layout is a trap. If a relative blocks the door, there is no second exit for the medical staff to escape.”
3. Psychological and Occupational Sequelae	Acute stress reactions	Immediate physiological and cognitive disruption after violent events.	“My hands trembled for hours. I couldn’t hold the syringe properly.” “After the assault, I had to go to the bathroom and cry just to ‘reset’ before my next procedure.” “I found myself trembling during an arterial line insertion. The trauma physically interferes with my ability to treat patients.”
	Moral injury and professional cynicism	Feeling betrayed or unsupported by institutions; loss of trust in leadership or public appreciation.	“After years of working through the pandemic, even neighbors treat us like threats. That hurts more than words.” “I feel like a traitor to my own wellbeing for staying in a system that clearly does not value my safety.” “I used to love this profession; now I wonder why I spent years studying just to be treated like a target.”
	Defensive practice and overcompensation	Excessive testing or documentation to protect against accusations or violence.	“To avoid complaints, we order every test possible. It’s a waste but we can’t risk another fight.” “I document every minor interaction in the charts primarily to protect myself from the next inevitable complaint.”
	Spillover into home life	Emotional exhaustion affecting family and personal interactions.	“At home I keep silent; I can’t listen to anyone talking. My head is full.” “I come home and sit in silence. I have no patience left for my own children after surviving the hostility of the shift.” “The anger I suppress at work inevitably leaks out at home. My family pays the price for the violence I face at the hospital.”
4. Coping, Containment, and Informal Safety Nets	Peer debriefing and collegial validation	Emotional ventilation and reassurance through informal discussion with colleagues.	“Only someone who’s been through it can calm you down.” “The locker room is our only therapy.” “We don’t talk to therapists; we talk to each other. That shared trauma is the only thing that keeps us sane.”
	Boundary-setting & redirection	Strategies to de-escalate or redirect aggression toward formal channels.	“I tell them to file a complaint if they want. That’s better than shouting back.” “I’ve started telling relatives: ‘I will pause this conversation until you can speak without shouting.’ You have to draw a line.” “I maintain a professional coldness now. It’s a defense mechanism to prevent emotional escalation.”
	Personal rationalization and normalization	Cognitive self-justification to cope with distress and helplessness.	“You say to yourself, the system is broken, anyone would fail here. That’s how you survive.” “I tell myself they are just grieving. It’s a lie I use so I don’t walk out of the door and never come back.”
5. Expectations of Support and System Obligations	Psychological, legal, and managerial triage	Desire for rapid, coordinated support following incidents—mental-health, legal, and administrative.	“We need a psychologist, a lawyer, and a chief who actually stands by us, not just paperwork later.” “The legal support is zero. If you file a complaint, you are essentially on your own against the perpetrator.” “The hospital offers no psychological counseling for victims. They expect you to be back at the bedside the next morning.”
	Security and access control	Preventive measures such as visitor screening, metal detectors, or trained guards.	“Hospitals need security like airports. Everyone should pass through control.” “We need X-ray machines and restricted access. Until then, we are just sitting ducks in a high-stress environment.” “Security guards are often told not to intervene. They are there for the building, not for the doctors.”
	Institutional acknowledgment and just culture	Recognition that violence is systemic and must be openly reported without blame.	“If incidents were discussed like medical errors, we could learn instead of hiding.” “The chief physician’s office doesn’t even send a ‘get well soon’ message. Our trauma is treated as an administrative nuisance.”
6. Pandemic-Specific Intensifiers	Resource scarcity and rule volatility	PPE shortages, changing policies, and uncertainty aggravate frustration among staff and public.	“One day bronchoscopy is forbidden, next day mandatory. These shifts create chaos and anger.” “During the pandemic, there was no equipment but plenty of anger. We were blamed for systemic shortages we couldn’t control.”
	Public distress and stigma	Hostility from society toward healthcare workers due to misinformation or fear.	“People avoided us in the elevator, like we carried the virus forever.” “The same people who clapped for us on balconies were the ones screaming at us in the corridors a month later.” “COVID turned the public’s fear into a weapon. The applause was temporary; the resentment felt permanent.”

## Data Availability

Data is contained within the article or Supplementary Materials. The original contributions presented in this study are included in the article/Supplementary Materials. Further inquiries can be directed to the corresponding author.

## Supplementary Materials

The following supporting information can be downloaded at: https://www.mdpi.com/article/10.3390/jcm15041503/s1, File S1: COREQ Checklist.

## Abbreviations

The following abbreviations are used in this manuscript:

SVP	Second Victim Phenomenon
VVP	Violence Victim Phenomenon

## References

[1] World Health Organization (WHO) Preventing Violence Against Health Workers. https://www.who.int/activities/preventing-violence-against-health-workers.

[2] Liu J., Gan Y., Jiang H., Li L., Dwyer R., Lu K., Yan S., Sampson O., Xu H., Wang C. (2019). Prevalence of workplace violence against healthcare workers: A systematic review and meta-analysis. Occup. Environ. Med..

[3] Vento S., Cainelli F., Vallone A. (2020). Violence Against Healthcare Workers: A Worldwide Phenomenon with Serious Consequences. Front. Public Health.

[4] Hahn S., Müller M., Hantikainen V., Kok G., Dassen T., Halfens R.J.G. (2012). Risk factors associated with patient and visitor violence in general hospitals: Results of a multiple regression analysis. Int. J. Nurs. Stud..

[5] Spelten E., van Vuuren J., O’Meara P., Thomas B., Grenier M., Ferron R., Helmer J., Agarwal G. (2022). Workplace violence against emergency health care workers: What Strategies do Workers use?. BMC Emerg. Med..

[6] Wu W. (2000). Medical error: The second victim. Br. Med. J. BMJ.

[7] Mira J.J., Carrillo I., Gil-Hernández E., Strametz R., Knežević Krajina H., Schrøder K., Tella S., Paiva S.G., Knežević B., Panella M. (2025). Key elements for designing effective second victim support interventions: A focus group study in European clinical settings. BMJ Open.

[8] Ramzi Z.S., Fatah P.W., Dalvandi A. (2022). Prevalence of Workplace Violence Against Healthcare Workers During the COVID-19 Pandemic: A Systematic Review and Meta-Analysis. Front. Psychol..

[9] Zhao S., Liu H., Ma H., Jiao M., Li Y., Hao Y., Sun Y., Gao L., Hong S., Kang Z. (2015). Coping with workplace violence in healthcare settings: Social support and strategies. Int. J. Environ. Res. Public Health.

[10] Sari H., Yildiz İ., Baloğlu S.Ç., Özel M., Tekalp R. (2023). The frequency of workplace violence against healthcare workers and affecting factors. PLoS ONE.

[11] Park S., Thrul J., Cooney E.E., Atkins K., Kalb L.G., Closser S., McDonald K.M., Schneider-Firestone S., Surkan P.J., Rushton C.H. (2024). Betrayal-Based Moral Injury and Mental Health Problems Among Healthcare and Hospital Workers Serving COVID-19 Patients Betrayal-Based Moral Injury and Mental Health Problems. J. Trauma Dissociation.

[12] Burns C.J., Marzoughi M., Sinko L., Saddawi-Konefka D., Stratton K., Sigakis M., Wixson M., Simmons S., Ramirez M., Kheterpal S. (2025). “The Pain and Suffering Are Just Too Great for Me to Manage”: A Qualitative Study of Emotional Distress among Anesthesiologists after Challenging Clinical Events. Anesthesiology.

[13] Krueger R., Casey M.A. (2000). Focus Groups: A Practical Guide for Applied Research.

[14] Ahmed S.K., Mohammed R.A., Nashwan A.J., Ibrahim R.H., Abdalla A.Q., Ameen B.M.M., Khdhir R.M. (2025). Using thematic analysis in qualitative research. J. Med. Surg. Public Health.

[15] Downe-Wamboldt B. (1992). Content analysis: Method, applications and issues. Health Care Women Int..

[16] Galtung J. (1969). Violence, peace, and peace research. J. Peace Res..

[17] Litz B.T., Stein N., Delaney E., Lebowitz L., Nash W.P., Silva C., Maguen S. (2009). Moral injury and moral repair in war veterans: A preliminary model and intervention strategy. Clin. Psychol. Rev..

[18] Anderson A., West S.G. (2011). Violence against mental health professionals: When the treater becomes the victim. Innov. Clin. Neurosci..

[19] Bhatti O.A., Rauf H., Aziz N., Martins R.S., Khan J.A. (2021). Violence against healthcare workers during the COVID-19 pandemic: A review of incidents from a lower-middle-income country. Ann. Glob. Health.

[20] Lafta R., Qusay N., Mary M., Burnham G. (2021). Violence against doctors in Iraq during the time of COVID-19. PLoS ONE.

[21] Belbase P., Basnet A., Parajuli A., Paudel S., Pandey A. (2021). Ordinance on the Safety and Security of Health Workers and Health Institutions in Nepal: A Critical Analysis. J. Nepal Health Res. Counc..

[22] World Health Organization, International Labour Organization Caring for Those Who Care: Guide for the Development and Implementation of Occupational Health and Safety Programmes for Health Workers.

[23] Schablon A., Wendeler D., Kozak A., Nienhaus A., Steinke S. (2018). Prevalence and Consequences of Aggression and Violence towards Nursing and Care Staff in Germany-A Survey. Int. J. Environ. Res. Public Health.

[24] Hines S.E., Chin K.H., Glick D.R., Wickwire E.M. (2021). Trends in moral injury, distress, and resilience factors among healthcare workers at the beginning of the COVID-19 pandemic. Int. J. Environ. Res. Public Health.

