# Supplementary material for: Defining the Violence Victim Phenomenon: A Qualitative Study Among Anesthesiology and Intensive Care Specialists
Source: J Clin Med. 2026 Feb 14;15(4):1503. doi: 10.3390/jcm15041503 (PMC12942364; doi:10.3390/jcm15041503)
Supplement: Supplementary file 1 [file jcm-15-01503-s001.zip › jcm-4094417-supplementary.pdf]

File S1. COREQ checklist (Defining the Violence Victim Phenomenon: A Qualitative Study Among Anesthesiology and Intensive Care Specialists)

Item No	Guide Questions/Description	Reported on Page #
Domain 1: Research team and reflexivity		
Personal Characteristics		
1. Interviewer/ facilitator	Which author/s conducted the interview or focus group?	Pg 3
2. Credentials	What were the researcher's credentials? E.g., PhD, MD	Pg 3
3. Occupation	What was their occupation at the time of the study?	Pg 3
4. Gender	Was the researcher male or female?	N/A
5. Experience and training	What experience or training did the researcher have?	Pg 3
Relationship with participants		
6. Relationship established	Was a relationship established prior to study commencement?	Pg 3
7. Participant knowledge of the interviewer	What did the participants know about the researcher, e.g., personal goals, reasons for doing the research?	N/A
8. Interviewer characteristics	What characteristics were reported about the interviewer/facilitator, e.g., bias, assumptions, reasons and interests in the research topic?	N/A
Domain 2: study design		
Theoretical framework		
9. Methodological orientation and theory	What methodological orientation was stated to underpin the study, e.g., grounded theory, discourse analysis, ethnography, phenomenology, content analysis?	Pg 4
Participant selection		
10. Sampling	How were participants selected, e.g., purposive, convenience, consecutive, snowball?	Pg 3
11. Method of approach	How were participants approached, e.g., face-to-face, telephone, mail, email?	Pg 3

Item No	Guide Questions/Description	Reported on Page #
12. Sample size	How many participants were in the study?	Pg 3
13. Non-participation Setting	How many people refused to participate or dropped out? Reasons?	N/A
14. Setting of data collection	Where was the data collected, e.g., home, clinic, workplace	Pg 3
15. Presence of nonparticipants	Was anyone else present besides the participants and researchers?	Pg 3
16. Description of sample	What are the important characteristics of the sample, e.g., demographic data, date?	Pg 3
Data collection		
17. Interview guide	Were questions, prompts, and guides provided by the authors? Was it pilot tested?	Pg 4
18. Repeat interviews	Were repeat interviews carried out? If yes, how many?	Pg 3
19. Audio/visual recording	Did the research use audio or visual recording to collect the data?	Pg 3
20. Field notes	Were field notes made during and/or after the interview or focus group?	Pg 3
21. Duration	What was the duration of the interviews or focus group?	Pg 3
22. Data saturation	Was data saturation discussed?	Pg 5
23. Transcripts returned	Were transcripts returned to participants for comment and/or correction?	N/A
Domain 3: analysis and findings		
Data analysis		
24. Number of data coders	How many data coders coded the data?	Pg 4
25. Description of the coding tree	Did the authors provide a description of the coding tree?	N/A
26. Derivation of themes	Were themes identified in advance or derived from the data?	Pg 5
27. Software	What software, if applicable, was used to manage the data?	Pg 5
28. Participant checking	Did participants provide feedback on the findings?	N/A
Reporting		

Item No	Guide Questions/Description	Reported on Page #
29. Quotations presented	Were participant quotations presented to illustrate the themes/findings? Was each quotation identified, e.g., participant number?	Pg 6-10 (Each quotation is not identified)
30. Data and findings consistent	Was there consistency between the data presented and the findings?	Pg 6-10
31. Clarity of major themes	Were major themes clearly presented in the findings?	Pg 6-10
32. Clarity of minor themes	Is there a description of diverse cases or a discussion of minor themes?	N/A
